# Phylogenetic analyses of *Chilomastix* and *Retortamonas* species using *in vitro* excysted flagellates

**DOI:** 10.1590/S1984-29612023070

**Published:** 2023-12-04

**Authors:** Jun Suzuki, Sanjib Kumar Sardar, Ajanta Ghosal, Naoko Yoshida, Hanako Kurai, Yudai Alex Takahashi, Yumiko Saito-Nakano, Sandipan Ganguly, Seiki Kobayashi

**Affiliations:** 1 Division of Clinical Microbiology, Department of Microbiology, Tokyo Metropolitan Institute of Public Health, Shinjuku-ku, Tokyo, Japan; 2 Division of Parasitology, National Institute of Cholera and Enteric Diseases, Belaghata, Kolkata, India; 3 Department of Tropical Medicine and Parasitology, School of Medicine, Juntendo University, Bunkyo-ku, Tokyo, Japan; 4 Division of Infectious Diseases, Shizuoka Cancer Center, Sunto-gun, Shizuoka, Japan; 5 Department of Life Science and Technology, Tokyo Institute of Technology, Meguro-ku, Tokyo, Japan; 6 Department of Parasitology, National Institute of Infectious Diseases, Shinjuku-ku, Tokyo, Japan

**Keywords:** **:** Excystation, Chilomastix mesnili, *Retortamonas* spp., Macaca fusucata, Urva auropunctata, phylogenetic analysis, **:** Excistação, Chilomastix mesnili, *Retortamonas* spp., Macaca fusucata, Urva auropunctata, análise filogenética

## Abstract

*In vitro* excystation of cysts of microscopically identified *Chilomastix* *mesnili* and *Retortamonas* sp. isolated from Japanese macaque*s* and *Retortamonas* sp. isolated from small Indian mongooses could be induced using an established protocol for *Giardia intestinalis* and subsequently by culturing with H_2_S-rich Robinson’s medium supplemented with *Desulfovibrio desulfuricans*. Excystation usually began 2 h after incubation in Robinson’s medium. DNA was isolated from excysted flagellates after 4 h of incubation or from cultured excysted flagellates. Phylogenetic analysis based on their 18S rRNA genes revealed that two isolates of *C*. *mesnili* from Japanese macaques belonged to the same cluster as a *C. mesnili* isolate from humans, whereas a mammalian *Retortamonas* sp. isolate from a small Indian mongoose belonged to the same cluster as that of an amphibian *Retortamonas* spp. isolate from a ‘poison arrow frog’ [sequence identity to AF439347 (94.9%)]. These results suggest that the sequence homology of the 18S rRNA gene of the two *C. mesnili* isolates from Japanese macaques was similar to that of humans, in addition to the morphological similarity, and *Retortamonas* sp. infection of the amphibian type in the small Indian mongoose highlighted the possibility of the effect of host feeding habitats.

## Resumo

Excistação *in vitro* de cistos de *Chilomastix mesnili* e *Retortamonas* sp. identificados microscopicamente, isolados de macacos japoneses e *Retortamonas* sp. isolados de um pequeno mangusto indiano podem ser induzidos usando um protocolo estabelecido para *Giardia intestinalis* e, subsequentemente por cultura com meio de Robinson rico em H_2_S suplementado com *Desulfovibrio desulfuricans*. A excistação geralmente começou 2 h após a incubação em meio de Robinson. O DNA foi isolado de flagelados excistados após 4 h de incubação ou de flagelados excistados cultivados. A análise filogenética baseada em seus genes 18S rRNA revelou que dois isolados de *C. mesnili* de macacos japoneses pertenciam ao mesmo grupo que um isolado de *C*. *mesnili* de um humano, enquanto um isolado de *Retortamonas* sp. de um mamífero, o pequeno mangusto indiano, pertencia ao mesmo agrupamento do isolado de *Retortamonas* spp. do anfíbio, ’sapo ponta-de-flecha’ [identidade sequencial para AF439347 (94,9%)]. Esses resultados sugeriram que a homologia sequencial do gene 18S rRNA dos dois isolados de *C*. *mesnili* de macacos japoneses era semelhante a de um humano, além da semelhança morfológica, e a infecção de *Retortamonas* sp. do tipo anfíbio ao pequeno mangusto indiano destacou a possibilidade de ser afetado pelo habitat alimentar de seu hospedeiro.

## Introduction

Retortamonadida (retortamonads) are a group of bacterivorous metamonads that produce rigid pyriform cysts and are currently represented by the single family Retortamonadidae, comprising two genera, *Chilomastix* and *Retortamonas* (Kulda et al., 2017). These metamonads live predominantly as intestinal endocommensals in both vertebrates and invertebrates (Kulda et al., 2017). However, two species have been reported as potential pathogens that cause diarrhoea in humans (*Chilomastix* *mesnili*) or unadapted avian hosts (*C. gallinarum*) (Boeck et al., 1921; Cepicka et al., 2008; Kulda et al., 2017). Among nonhuman primates, *C. mesnili* infection cases have also been reported (Levecke et al., 2007); However, genetic information on *C. mesnili* isolated from nonhuman primates is extremely limited (Jeong et al., 2019). *C. mesnili*-infected nonhuman primates are often difficult to morphologically distinguish from infected humans. However, Hegner (1924) reported that *Chilomastix* sp. cysts from Geoffroy’s spider monkey (*Ateles geoffroyi* Kuhl) resembled those of *C. mesnili* isolated from humans in shape, although they were considerably larger. The results of phylogenetic analyses of *Chilomastix* spp. and *Retortamonas* spp., based on 18S rDNA, suggested that Retortamonadida had tendencies of non-monophyly and high genetic diversity of the genus *Chilomastix* (Cepicka et al., 2008).

In the present study, we attempted to determine the phylogenetic position of morphologically identified *C. mesnili* and *Retortamonas* sp. isolated from a Japanese macaque (*Macaca fusucata*: a nonhuman primate and mainly vegetarian) and determine the effect of feeding habitat on infective *Retortamonas* sp. species in a small Indian mongoose (*Urva auropunctata*), which habitually feeds on frogs that may be infected with a different phyletic *Retortamonas* sp. from that of mammalian hosts.

To obtain valid results from the first genetic analysis of a protozoan parasite and determine the cause of genetic diversity, such as in the genus *Chilomastix*, establishing a culture system to maintain the protozoan species continuously is necessary. Before establishing culture isolates and performing phylogenetic analyses, an *in vitro* excystation system for *C. mesnili* and *Retortamonas* spp. using cyst-positive samples was established. This is because treatment with the low-pH induction solution in the *in vitro* excystation protocol using partially purified cysts could be eliminated, often contaminating and easily isolating non-cyst-forming parasitic and coprozoic flagellates into the medium from stool samples of Japanese macaques and small Indian mongooses. Another reason is that, even when the culture isolate could not be established, DNA samples from the excysted flagellates could be obtained directly in a short period without cultivation. In the present study, culture isolates of *Retortamonas* spp. were established and maintained for over 3 years in Robinson’s medium (Robinson, 1968) supplemented with *Desulfovibrio desulfuricans* (Yoshida et al., 2019). However, a continuous culture system for *C. mesnili* using motile flagellates excysted from cysts in stool samples of Japanese macaques could not be established using established culture media [Robinson’s medium (Robinson, 1968), Balamuth’s medium (Balamuth, 1946), TYSGM-9 (Diamond, 1982), and modified TYI-S-33 medium (Keister, 1983)]. Through this culture trial, an excystation protocol and a short-term culture system using an egg yolk medium (Balamuth’s medium) were established by supplementing with mucin and mucin-degradable *Bifidobacterium longum*.

Subsequently, we used the established excystation protocol (Boucher & Gillin, 1990) for *Giardia lamblia* (syn., *G. intestinalis*) by culturing with Robinson’s medium supplemented with *D*. *desulfuricans*. In addition, we designed *C. mesnili* and *Retortamonas* sp*.* specific primers for phylogenetic analyses based on the genomic sequences of 18S rRNA genes that were revealed using culture isolates and eukaryote-specific primers (Silberman et al., 2002; Cepicka et al., 2008).

## Materials and Methods

### Obtaining cysts

Stool samples from 213 wild Japanese macaques were collected from monkey trails near villages close to mountains at three different places in the Chubu and Kanto regions, Japan, where they were usually populated and obtained food, and from a small wild Indian mongoose obtained from a rural village in West Bengal, India, where it was commonly found. Consequently, 17 *Chilomastix mesnili* and 6 *Retortamobas* sp. cyst-positive stool samples of wild Japanese macaques could be obtained and used for establishing those culture isolates.

*C. mesnili* cysts (MfOkutamaCm and MfChichibuCm) were obtained from *C*. *mesnili* cyst-positive stool samples of wild Japanese macaques (inhabiting Okutama, Tokyo, Japan and Chichibu, Saitama, Japan) and identified morphologically by microscopic stool examination. However, a sufficient number of cysts from isolates obtained four different samples of *Retortamonas* spp. (UaBengalR: from a small wild Indian mongoose inhabiting West Bengal, India; MfHotakaR1, R2, and R6: from wild Japanese macaques inhabiting Azumino, Nagano, Japan) for the excystation experiment were difficult to obtain from the cyst-positive stool samples. Nevertheless, every four culture isolates from each cyst-positive stool sample could be established using Robinson’s medium supplemented with *D*. *desulfuricans* and maintained at 35.5 °C, which exerts an excystation stimulatory effect on the cysts of *Entamoeba chattoni* (*E. polecki* subtype 2) isolated from Japanese macaques, *E. polecki* (subtype 1 and 3), *E. suis* and *Endolimax* sp. isolated from swine, and *Nyctotherus teleacus* isolated from a radiated tortoise. They could be subcultured using the same Robinson’s medium for over a year (Robinson, 1968; Yoshida et al., 2019; Suzuki et al., 2020). Under these culture conditions, the four *Retortamonas* spp. isolates spontaneously and continuously produced a sufficient number of cysts in the medium for over 3 years. The size of the culture flagellates of these isolates was approximately 2–3-fold the size of the cysts produced. The cysts of UaBengalR and MfHotakaR1, R2, and R6 were obtained by culturing these isolates and used as cyst samples for *in vitro* excystation experiments.

### Partial purification of cysts

Cyst-positive stool samples were suspended in 10 times the volume of high-purity water in 50-mL plastic centrifuge tubes and left to stand for a few minutes to allow the precipitation of large debris. Thereafter, the supernatants were transferred into new plastic tubes using a transfer pipette (volume: 3 mL, drops per mL: 22) to further remove debris which could not pass through the aperture of the pipette. Next, the supernatants were stored at 4 °C with suspension in high-purity water after washing via centrifugation (275 × *g* for 5 min twice) with 10 times the volume of high-purity water and used within a few weeks.

Before excystation, the cysts in these suspensions (2 mL) were partially purified using the Percoll density gradient centrifugal separation method (400 × *g*, 20 min) with 4 mL of 40 and 100% Percoll PLUS (GE Healthcare Bio-Sciences AB, Little Chalfont, UK) in a 50-mL plastic tube. The cyst-rich samples of *C*. *mesnili* and four *Retortamonas* spp. were collected into 10 times the amount of high-purity water from the border fraction, washed twice with high-purity water via centrifugation (275 × *g* for 5 min), and stored at 4 °C until use.

### Excystation and harvest of excysted flagellates

The established excystation protocol for *G. lamblia* (syn., *G. intestinalis*) cysts (Boucher & Gillin, 1990) that employs a low-pH induction solution (pH 4.0) consists of solutions of L-cysteine hydrochloride and reduced glutathione and is incubated with cysts at 38 °C for 30 min (step 1). After washing with Hanks’ solution (pH 7.4 ) via centrifugation (275 × *g* for 5 min) once, a digestive enzyme (chymotrypsin) solution (pH 8.0) was applied and incubated with the cysts at 38 °C for 30 min (step 2). Thereafter, the cyst fraction obtained via centrifugation (275 × *g* for 5 min) was transferred into H_2_S-rich Robinson’s medium supplemented with *D. desulfuricans* and incubated at 35.5 °C (step 3).

Percoll density gradient centrifugal separation and treatment with a low-pH induction solution in excystation (step 1) could eliminate coexisting non-cyst-forming parasitic flagellates, such as *Pentatrichomonas* spp., and coprozoic flagellates. However, these steps could not eliminate the cysts of parasitic flagellate species, such as *Enteromonas* sp., in the case of coexistence in stool samples of Japanese macaques and small Indian mongooses.

Excysted motile flagellates of the two *C. mesnili* and four *Retortamonas* spp. isolates usually began to appear 2 h after incubation in H_2_S-rich Robinson’s medium (excystation step 3). Excysted motile flagellates of *C. mesnili* (MfOkutamaCm and MfChichibuCm) in the medium were harvested after 4 h of incubation via centrifugation (275 × *g* for 5 min). The number of motile *C. mesnili* flagellates gradually decreased after excystation and disappeared 24–48 h after incubation in Robinson’s medium. The incubation time required to obtain the highest number of motile *C. mesnili* flagellates was 3–5 h (e.g. when the initial concentration of cysts was 5–6 × 10^4^/mL, approximately 2–3 × 10^4^/mL motile flagellates usually appeared after 4 h of incubation). One portion of the excysted flagellates of *C. mesnili* was inoculated into Balamuth’s medium supplemented with mucin and *Bifidobacterium longum,* and grown flagellates were harvested. Excysted flagellates of the four *Retortamonas* spp. isolates were harvested after 48–72 h of incubation. This was because the cells were efficiently grown in H_2_S-rich Robinson’s medium and could be cultured continuously. It was also confirmed that the size and morphological features of spontaneously produced cysts in the medium before and after the excystation experiment were the same.

### Culture media

Robinson’s medium supplemented with 10% heat-inactivated adult bovine serum, *Escherichia coli* (DH5α), *D*. *desulfuricans*, and starch (rice powder) (Robinson, 1968; Yoshida et al., 2019) was used to culture *Retortamonas* spp. isolates and as excystation medium (step 3) for *Chilomastix* sp. and *Retortamonas* spp cysts. The sulfate-reducing anaerobic bacterium *D. desulfuricans* (NBRC 13699, NITE Biological Resource Center, Chiba, Japan) was cocultured with *E. coli* (type B: DH5α) in a modified ATCC 207 medium (Yoshida et al., 2019).

Balamuth’s medium containing egg yolk extract (Balamuth, 1946) (4 mL) supplemented with 4% heat-inactivated adult bovine serum, 0.2 mL/mL of modified ATCC 207 medium, including mucin (FUJIFILM Wako Pure Chemical Corp., Osaka, Japan) and mucin-degradable *B*. *longum* (No. 114370; Nite Biological Resource Center, Chiba, Japan), cocultured with *E. coli* (DH5α) and *D. desulfuricans* (NBRC 13699) in screw-capped glass tube (13 × 100 mm) were used as culture media for *C. mesnili* (MfOkutamaCm and MfChichibuCm). Mucin was sterilised by autoclaving at 121 °C for 15 min. *B. longum* was cultured anaerobically using TOS propionate agar medium (Yakult Pharmaceutical Industry Co., Ltd., Tokyo, Japan) and then inoculated into modified ATCC207 medium (Yoshida et al., 2019) cocultured with *E. coli* (DH5α) and *D. desulfuricans* containing mucin (3.5 mg/mL) at 35.5 °C for 2 days. The mucin- *B. longum* supplement was stored at 4 °C and used within 2 weeks.

Mucin was used because of its growth-promoting effect on some intestinal protozoan parasites, such as *Entamoeba histolytica*, in TYGM9 culture medium (Diamond, 1982). Balamuth’s medium was developed to establish a culture isolate of *C. mesnili* from Japanese macaques for a more reliable phylogenetic analysis.

### Sequence and phylogenetic analyses

DNA isolated from the excysted and cultured flagellates of *C. mesnili* (MfOkutamaCm and MfChichibuCm) and *Retortamonas* spp. (UaBengalR, MfHotakaR1, R2, and R6) (500–10,000 flagellate cells), were used as templates for PCR amplification of 18S ribosomal RNA (rRNA) genes. DNA was extracted from harvested flagellates using a Cica Geneus Total DNA Prep Kit (Kanto Chemical Co., Chuo-ku, Tokyo, Japan). Primers for *C. mesnili* [(Mnil-F1:5′- TCGAGCATATATTAAAGTTGTTGCGT-3′) and (Mnil-R1:5′- ACAGAGGTGTCTGATCTCCTT-3′)] and *Retortamonas* spp. [(Ret-F1:5′-AAGGATGGCAGCAGGCGCGAAA-3′) and (Ret-R1:5′- TCCCCTGGCTTTCGATCTTGA-3′)] were designed based on the genomic sequences of reference strains of *C. mesnili* (NOVA: KC960589) isolated from a human and *Retortamonas* sp. (ATCC50375: AF439347) isolated from a ‘poison arrow frog’. The following cycling parameters were used: initial denaturation at 94 °C for 5 min; 35 cycles of 94 °C for 30 s, 58 °C for 30 s, and 72 °C for 1 min; and a final extension at 72 °C for 5 min.

PCR products were directly sequenced using an ABI Prism BigDye Terminator v3.1 cycle sequencing ready reaction Kit on an ABI Prism 3500 Genetic Analyser (Applied Biosystems Japan, Ltd., Tokyo, Japan). Multiple alignments and phylogenetic analyses of the 18S rRNA gene sequences of *C. mesnili* and *Retortamonas* spp. isolates were performed using ClustalW and the maximum-likelihood (ML) method in MEGA software version 6 (Ronquist et al., 2012; Tamura et al., 2013). The ML tree was derived using the Kimura 2-parameter model, employing estimates of the gamma distribution with five rate categories. Statistical significance was evaluated by bootstrapping with 1,000 replicates. The ML tree data files were visualised using MEGA version 6.

The two PCR primer sets for *C. mesnili* and *Retortamonas* spp. also confirmed that the primer sets did not amplify the DNA extracted from the excysted flagellates of the microscopically identified *Enteromonas* sp. isolated from Japanese macaques.

**Figure 1:**
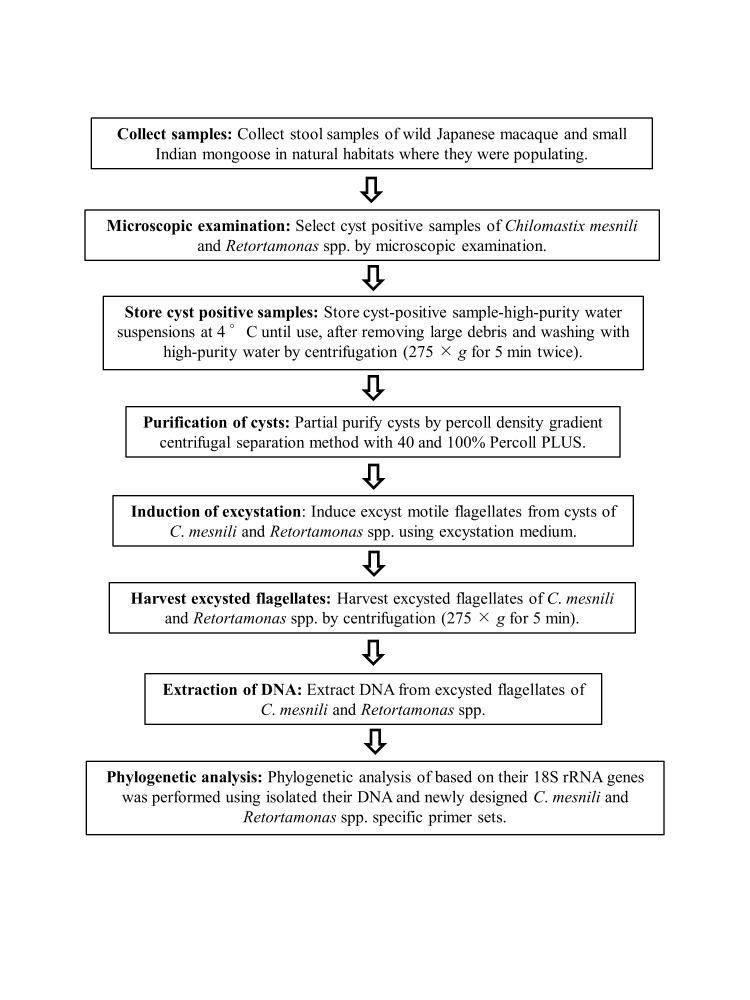
Flowchart of the approach to phylogenetic analysis in the present study.

A flowchart of the phylogenetic analysis approach used in the present study is shown in Figure 1.

## Results

**Figure 2:**
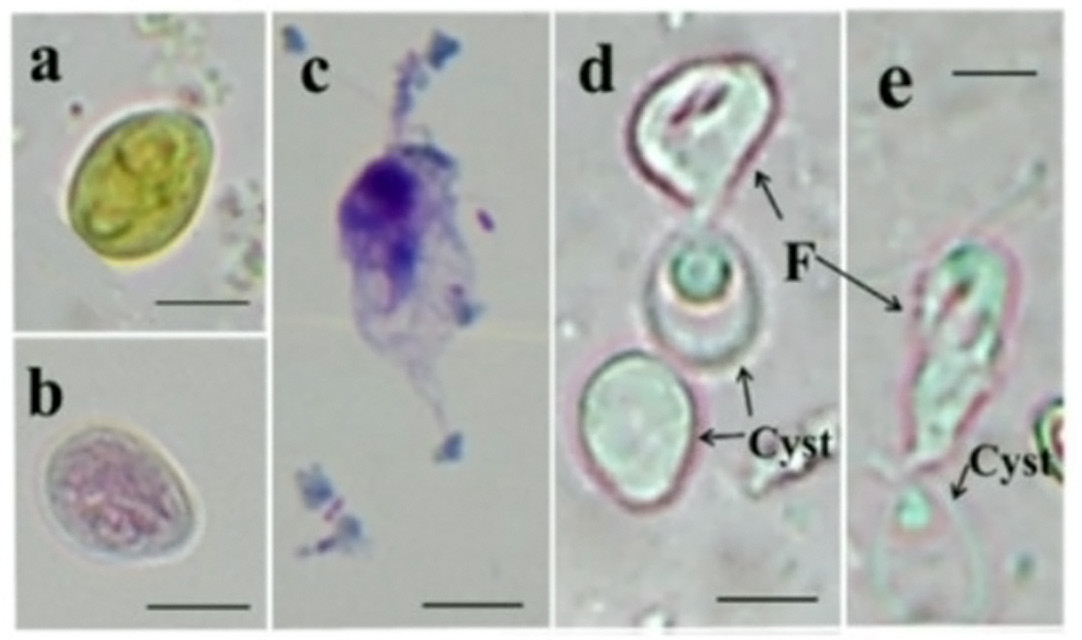
Light microscopy images of the cysts and flagellates of *Chilomastix mesnili* (MfOkutamaCm). (a, b, c, d and e) *C. mesnili* (MfOkutamaCm); (a) Cysts stained using an iodine stain. (b) Cysts stained using a Trichrome stain (Wheatley, 1951). (c) Flagellates stained using a Giemsa stain. (d and e) Excystation process of *C. mesnili* (MfOkutamaCm). Scale bar = 5 μm; F: flagellate. Approximate sizes of cysts: *C. mesnili* (MfOkutamaCm) [length (L) × breadth (B): 9.4–11.3 × 6.6–7.8 μm]. Approximate sizes of flagellates: *C. mesnili* (MfOkutamaCm, L × B: 12.5–17.8 × 4.4–7.6 μm).

The excysted flagellates of the *C. mesnili* (MfOkutamaCm) and *Retortamonas* sp. (MfHotakaR1) isolates are shown in Figure 2d and e. The cysts of *C. mesnili* (MfOkutamaCm) from the Japanese macaque measured 9.4–11.3 μm in length and 6.6–7.8 μm in breadth (see Figure 2 legend). *C. mesnili* infecting Japanese macaques (a nonhuman primate) (Figure 2a, b, and c) is often difficult to distinguish morphologically from *C. mesnili* infecting humans. However, the size of the cysts of *C. mesnili* from humans differed from that from Japanese macaques. In our results, the cysts of another *C. mesnili*-infected Japanese macaque (MfChichibuCm) from a different populating area showed a tendency to be larger, mainly in breadth, than those from humans (7.92–9.9 and 6.93–7.92 μm in length and breadth, respectively).

Continuous culture of the excysted flagellates of *C. mesnili* (MfOkutamaCm and MfChichibuCm) confirmed the proliferation of flagellates in Balamuth’s medium supplemented with mucin and *B*. *longum*. They could survive for up to 13 days, although the culturable period was still short. However, this result demonstrates the possibility of establishing a culture of *C. mesnili* isolated from Japanese macaques.

**Figure 3:**
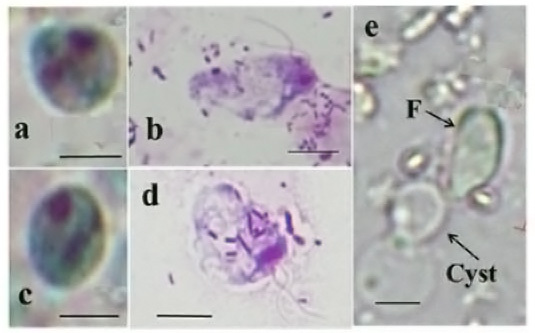
Light microscopy images of the cysts and flagellates of *Retortamonas* spp. (UaBengalR and MfHotakaR1). (a and b): *Retortamonas* sp. (UaBengalR); (c, d, and e): *Retortamonas* sp. (MfHotakaR1). (a and c) Cysts stained using a Trichrome stain (Wheatley, 1951). (b and d) Flagellates stained using a Giemsa stain. (e) Excystation process of *Retortamonas* sp. (MfHotakaR1). Scale bar = 5 μm; F: flagellate. Approximate sizes of cysts: *Retortamonas* sp. (UaBengalR) [length (L) × breadth (B): 3.9–5.6 × 3.0–4.7 μm]; *Retortamonas* sp. (MfHotakaR1, L × B: 5.2–6.6 × 4.0–5.5 μm). Approximate sizes of flagellates: *Retortamonas* sp. (UaBengalR, L × B: 12.2–16.0 × 3.3–7.7 μm); *Retortamonas* sp. (MfHotakaR1, L × B: 9.1–10.2 × 4.1–5.3 μm).

Notably, we found that the size of *Retortamonas* spp. flagellates (UaBengalR) was notably larger (approximately 1.5 times) than that of MfHotakaR1, although the cysts of both isolates were approximately of the same size (Figure 3a, b, c, d, and e, and their measured values are described in the figure legend).

**Figure 4:**
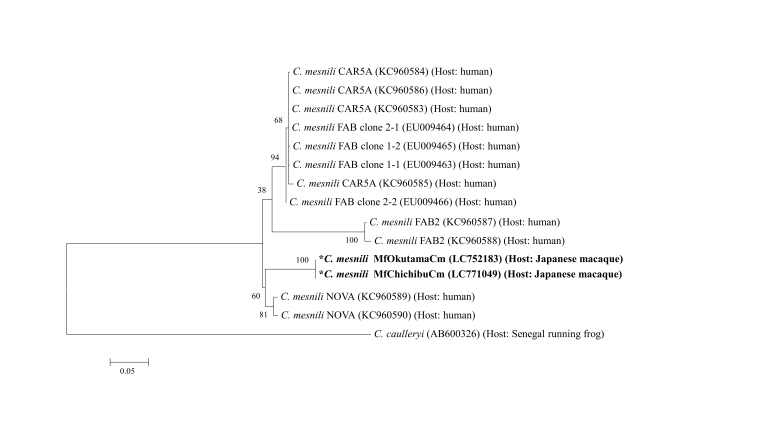
Phylogenetic analysis of *Chilomastix mesnili* (MfOkutamaCm and MfChichibuCm) isolates based on their 18S rRNA gene sequences. Maximum likelihood (ML) tree of *C. mesnili* based on 18S rRNA sequences. An ML tree was derived using the Kimura-2 parameter model. Significant bootstrap support (>500) from 1,000 replicates is indicated on the left of the supported nodes. The scale bar represents the evolutionary distance based on the number of changes per site. The numbers in parentheses represent GenBank accession numbers. *C. mesnili isolates from Japanese macaques in the present study.

**Figure 5:**
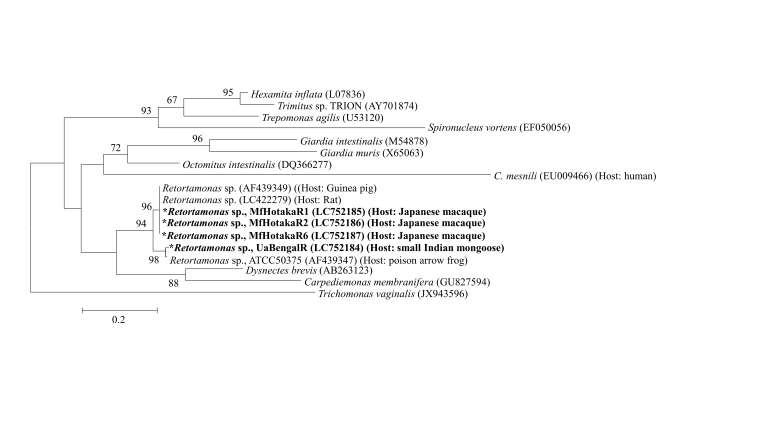
Phylogenetic analysis of *Retortamonas* spp. (UaBengalR and MfHotakaR1, R2, and R6) isolates based on their 18S rRNA gene sequences. Maximum likelihood (ML) tree of *Retortamonas* spp. and those of *Fornicata* parasites based on 18S rRNA sequences. Phylogenetic analysis of *Retortamonas* spp. was performed as described in Figure 4. *Retortamonas spp. isolated from Japanese macaques and small Indian mongooses.

Phylogenetic analysis of the 18S rRNA gene of *C. mesnili* [MfOkutamaCm (LC752183) and MfChichibuCm (LC771049)] revealed that it belonged to the same cluster as that of *C. mesnili* (NOVA: KC960589 and KC960590) from a human, as shown in Figure 4. The sequence identities of MfOkutamaCm (LC752183) and MfChichibuCm (LC771049) to a human isolate (NOVA: KC960590) were 83.0% (738/889 bp) and 82.7% (735/889 bp), respectively. The sequence identity of MfOkutamaCm (LC752183) and MfChichibuCm (LC771049) was 99.7% (875/878 bp). We also revealed that a *Retortamonas* spp. isolate (UaBengalR, LC752184) belonged to the same cluster as that of the isolate from the ‘poison arrow frog’ [sequence identity to AF439347 (ATCC 50375) was 94.9% (687/724 bp)] (Figure 5). In contrast, we noticed that the three *Retortamonas* spp. isolates [MfHotakaR1 (LC752185), MfHotakaR2 (LC752186), and MfHotakaR6 (LC752187), which showed 100% identity, belonged to the same cluster as that of the isolates from guinea pigs (AF439349) and rats (LC422279) [sequence identities of LC752185, LC752186, and LC752187 to AF439349 and LC422279 were 99.4% (687/691 bp) and 99.9% (688/689 bp), respectively]. As a reference, the sequence identities of three mammalian *Retortamonas* spp. isolates (MfHotakaR1, R2, and R6) from a Japanese macaque and one mammalian isolate (UaBengalR) from a small Indian mongoose were 79.1% (582/731 bp). 

## Discussion

To the best of our knowledge, this is the first study to show the genetic similarity of 18S rRNA between *C. mesnili* (NOVA: KC960589 and KC960590) isolated from humans and *C. mesnili* [MfOkutamaCm (LC752183) and MfChichibuCm (LC771049)] isolated from Japanese macaques, in addition to their morphological similarity. However, the cysts of *C. mesnili* in infected Japanese macaques appear larger than those in infected humans. This result is consistent Hegner (1924), who reported on the size of *Chilomastix* sp. cysts from Geoffroy’s spider monkey (*Ateles geoffroyi* Kuhl). They found that cysts in humans infected with *C. mesnili* ranged from 7 to 9 μm in length and from 4 to 6 μm in breadth, while those from Geoffroy’s spider monkey measured from 8.47 to 10.26 μm in length and from 5.92 to 7.62 μm in breadth. The reason for the difference in cyst size between humans and nonhuman primates [Japanese macaque and Geoffroy’s spider monkey (Hegner, 1924)] is unclear. However, differences in cyst size may be affected by flagellate size. The motile flagellates of *C. mesnili* in humans display a wide range of sizes, ranging from 3 to 19 μm in length and 2 to 9 μm in width (Boeck, 1921). However, in the case of *C. mesnili* from Japanese macaques (MfOkutamaCm and MfChichibuCm), no motile flagellates <10 μm in length [e.g., 12.5–17.8 μm in length and 4.4–7.6 μm in width (MfOkutamaCm)] were observed.

In the culture process, we also showed that mucin and *B. longum* exerted growth-promoting effects on *C. mesnili* (MfOkutamaCm and MfChichibuCm). *Bifidobacterium* species can colonise the intestinal mucosa through their adhesion proteins (Nishiyama et al., 2020) and their ability to degrade mucin (Ruas-Madiedo et al., 2008), which is the principal component of mucus in the mucosa. *Bifidobacterium* species (*B. animales*, B-12 strain), other than *B. longum*, exerted the same growth-promoting effect as mucin in *C. mesnili* (MfOkutamaCm) (data not shown). These bacteria inhabit the gut of primates (Modrackova et al., 2021). These results suggest that *C. mesnili* (MfOkutamaCm and MfChichibuCm) secretes an adhesin-like substance that adheres to the intestinal mucosa, such as some intestinal protozoan parasites that cause diarrhoea [*Tritrichomonas foetus* (Tolbert et al., 2013) and *E. histolytica* (Mann et al., 1991)].

The phylogenetic analysis of Retortamonadida and genus *Chilomastix,* based on 18S rDNA, first reported by Cepicka et al. (2008), suggested that Retortamonadida had tendencies of non-monophyly and high genetic diversity of the genus *Chilomastix.* Up to 2023, the 18S rRNA gene sequences of 12 DNA clones obtained from 4 isolates (CAR5A, FAB, FAB2, and NOVA) of *C. mesnili* have been registered to GenBank by Cepicka et al. (2008) and direct submitted and registered to GenBank by Cepicka in 2013. According to our phylogenetic analysis using the 12 18S rRNA gene sequences of *C. mesnili* from humans, it branched into 2 clusters: cluster A (CAR5A, FAB, and FAB2) and cluster B (NOVA) of *C. mesnili* isolates in the phylogenetic tree. In the present study, the 18S rRNA gene sequences of two *C. mesnili* isolates [MfOkutamaCm (LC752183) and MfChichibuCm (LC771049)] from Japanese macaque belonged to cluster B (NOVA) in the phylogenetic tree.

Silberman et al. (2002) reported that the identities among mammalian isolates ranged from 99.5 to 99.8%, whereas those among two amphibian isolates were slightly lower (96.5%), suggesting a tendency for 18S rRNA gene sequence homologies among *Retortamonas* spp. of mammalian and amphibian animals. Furthermore, they found that the overall identity between the mammalian and amphibian retortamonad 18S rRNA genes was 74.1%. We assumed that the high homology (94.9%) of the 18S rRNA gene sequences of *Retortamonas* spp. (UaBengalR, LC752184) with that of the isolate from the ‘poison arrow frog’ (AF439347) might be due to the feeding habits of the small Indian mongoose, which feeds on amphibians, insects, small mammalian animals, and reptiles. These results suggest that *Retortamonas* sp. infection may be affected by the feeding habitat of its host.

Based on the results, the flagellates excysted from cysts appeared to be reliable specimens, similar to culture isolates for the genetic analysis of cyst-forming parasitic flagellates, such as those belonging to Retortamonadidae and Enteromonadidae. Due to the high genetic diversity of the *Chilomastix* genus (Cepicka et al., 2008) and low but non-negligible genetic diversity even among *C. mesnili* species, and the limited knowledge of the genetic information on *C. mesnili* isolated from nonhuman primates, further genetic analyses using more culture isolates of *C*. *mesnili* from humans and nonhuman primates are necessary.

## Conclusion

The *in vitro* excystation of *Chilomastix* and *Retortamonas* species was induced. The excysted flagellates were reliable specimens for phylogenetic analysis. Novel insights into *C. mesnili* isolated from nonhuman primates were gained from genetic analyses.
